# Genome Sequences of Subcluster K5 Mycobacteriophages AlleyCat, Edugator, and Guillsminger

**DOI:** 10.1128/genomeA.01122-17

**Published:** 2017-11-09

**Authors:** Rodney A. King, Tina M. Slowan-Pomeroy, Jodi E. Thomas, Tithe Ahmed, Katie L. Alexander, James M. Biddle, Makenzie K. Daniels, Jenna R. Rowlett, Taylor E. Senay, Claire A. Rinehart, Amanda K. Staples, Naomi S. Rowland, Bobby L. Gaffney, Christine B. Emmons, Maya D. Hauk, Rebecca L. Nguyen, Leonard Naegele, Summer S. Strickland, Laura A. Briggs, Alexander N. Rush, Sanghamitra Saha, Rachna Sadana, Steven G. Cresawn, Daniel A. Russell, Rebecca A. Garlena, Welkin H. Pope, Deborah Jacobs-Sera, Graham F. Hatfull

**Affiliations:** a Department of Biology, Western Kentucky University, Bowling Green, Kentucky, USA; b Department of Biology, Truckee Meadows Community College, Reno, Nevada, USA; c Department of Biology, University of Housto-Downtown, Houston, Texas, USA; d Department of Biology, James Madison University, Harrisonburg, Virginia, USA; e Department of Biological Sciences, Pittsburgh, Pennsylvania, USA

## Abstract

Bacteriophages AlleyCat, Edugator, and Guillsminger were isolated on *Mycobacterium smegmatis* mc^2^155 from enriched soil samples. All are members of mycobacteriophage subcluster K5, with genomes of 62,112 to 63,344 bp. Each genome contains 92 to 99 predicted protein-coding genes and one tRNA. Guillsminger is the first mycobacteriophage to carry an IS*1380* family transposon.

## GENOME ANNOUNCEMENT

Bacteriophages are the most numerous biological entities in the biosphere and play large roles in bacterial diversity and ecology (1). The genomes of three double-stranded DNA (dsDNA)-tailed bacteriophages infecting *Mycobacterium smegmatis* mc^2^155, AlleyCat, Edugator, and Guillsminger, are reported here. Phage isolation and genome analysis were conducted by undergraduate students in the Howard Hughes Medical Institute’s Science Education Alliance Phage Hunters Advancing Genomics and Evolutionary Science (HHMI SEA-PHAGES) program (2). AlleyCat, Edugator, and Guillsminger were isolated from Kentucky, Texas, and Nevada soil samples, respectively, using standard enrichment techniques and incubation temperatures between 30°C and 37°C (1).

Electron microscopy shows that AlleyCat, Edugator, and Guillsminger are siphoviruses with 50- to 65-nm isometric heads and 150-nm noncontractile tails. Phage genomic DNA was purified and sequenced at the University of Pittsburgh using an Illumina MiSeq platform. Single-end run reads (140 to 150 bases) were assembled to generate major contigs with at least 2,061-fold coverage. The genomes of AlleyCat, Edugator, and Guillsminger are 62,112 bp and 63,344 bp and 63,153 bp long, respectively, and they all have 11-base 3′ single-stranded DNA extensions (right end, 5′-CTCAGTGGCAT-3′). The G+C content ranges from 65.3% (AlleyCat and Edugator) to 65% (Guillsminger), similar to that of *M. smegmatis* (67.4% G+C). Genome-wide BLAST analysis indicates that AlleyCat is most similar to subcluster K5 phages Larva and Edugator (98% and 97% span length matches, respectively), and Guillsminger is most similar to subcluster K5 phages Gengar and Waterfoul (91% span length matches).

Putative genes were identified using Glimmer (3), GeneMark (4), Aragorn (5), and tRNAscan-SE (6) and by manual inspection and annotation revision using DNA Master (http://cobamide2.bio.pitt.edu/computer.htm) and PECAAN (https://discover.kbrinsgd.org). Gene functions were assigned using BLASTP (7) and HHpred (8). AlleyCat, Edugator, and Guillsminger have 99, 92, and 94 genes, respectively, and each has a single tRNA gene; functions were predicted for 41 to 47% of the genes. Virion structure and assembly genes are located in the left part of the genome, followed by lysis cassettes that include lysin A (AlleyCat *30*, Edugator *29*, and Guillsminger *29*), lysin B (AlleyCat *31*, Edugator *30*, and Guillsminger *30*), and holin (AlleyCat *32*, Edugator *31*, and Guillsminger *31*) genes. The immunity cassettes, including serine integrases and immunity repressors, are centrally located, and all three phages are predicted to be temperate. Putative DNA metabolism and replication genes are located in the right arm, although a predicted DNA polymerase III (Pol III) gene (Larva *48* and Edugator *46*) is absent from AlleyCat. The rightmost thirds of the genomes vary considerably and are dominated by genes of unknown function.

All genes are transcribed in the forward direction, except for a gene (AlleyCat *38*, Guillsminger *38*, and Edugator *38*) encoding a 43-residue protein related to the C termini of several hydrolases, a gene of unknown function (AlleyCat *41*, Guillsminger *41*, and Edugator *40*), and the repressor (AlleyCat *42*, Guillsminger *42*, and Edugator *41*). Guillsminger is unusual in carrying an IS*1380* family transposon inserted near the right end of the genome. Although similar transposons are found in mycobacterial strains, this is the first IS*1380*-like insertion in over 1,300 sequenced mycobacteriophages.

### Accession number(s).

The GenBank accession numbers for AlleyCat, Edugator, and Guillsminger are MF185717, MF185719, and MF185720, respectively.

## References

[1] Pope WH, Bowman CA, Russell DA, Jacobs-Sera D, Asai DJ, Cresawn SG, Jacobs WR, Hendrix RW, Lawrence JG, Hatfull GF, Science Education Alliance Phage Hunters Advancing Genomics, Evolutionary Science, Phage Hunters Integrating Research and Education, Mycobacterial Genetics Course 2015 Whole genome comparison of a large collection of mycobacteriophages reveals a continuum of phage genetic diversity. Elife 4:e06416. 25919952 10.7554/eLife.06416 PMC4408529

[2] Jordan TC, Burnett SH, Carson S, Caruso SM, Clase K, DeJong RJ, Dennehy JJ, Denver DR, Dunbar D, Elgin SC, Findley AM, Gissendanner CR, Golebiewska UP, Guild N, Hartzog GA, Grillo WH, Hollowell GP, Hughes LE, Johnson A, King RA, Lewis LO, Li W, Rosenzweig F, Rubin MR, Saha MS, Sandoz J, Shaffer CD, Taylor B, Temple L, Vazquez E, Ware VC, Barker LP, Bradley KW, Jacobs-Sera D, Pope WH, Russell DA, Cresawn SG, Lopatto D, Bailey CP, Hatfull GF 2014 A broadly implementable research course in phage discovery and genomics for first-year undergraduate students. mBio 5:e01051-13. doi:10.1128/mBio.01051-13. 24496795 PMC3950523

[3] Delcher AL, Bratke KA, Powers EC, Salzberg SL 2007 Identifying bacterial genes and endosymbiont DNA with Glimmer. Bioinformatics 23:673–679. doi:10.1093/bioinformatics/btm009. 17237039 PMC2387122

[4] Besemer J, Borodovsky M 2005 GeneMark: web software for gene finding in prokaryotes, eukaryotes and viruses. Nucleic Acids Res 33:W451–W454. doi:10.1093/nar/gki487. 15980510 PMC1160247

[5] Laslett D, Canback B 2004 ARAGORN, a program to detect tRNA genes and tmRNA genes in nucleotide sequences. Nucleic Acids Res 32:11–16. doi:10.1093/nar/gkh152. 14704338 PMC373265

[6] Lowe TM, Eddy SR 1997 tRNAscan-SE: a program for improved detection of transfer RNA genes in genomic sequence. Nucleic Acids Res 25:955–964. doi:10.1093/nar/25.5.0955. 9023104 PMC146525

[7] Altschul SF, Gish W, Miller W, Myers EW, Lipman DJ 1990 Basic local alignment search tool. J Mol Biol 215:403–410. doi:10.1016/S0022-2836(05)80360-2. 2231712

[8] Söding J, Biegert A, Lupas AN 2005 The HHpred interactive server for protein homology detection and structure prediction. Nucleic Acids Res 33:W244–W248. doi:10.1093/nar/gki408. 15980461 PMC1160169

